# Supplementary material for: Are carbon emissions trading and green financial instruments synergistic? -Comprehensive quantitative research based on content analysis
Source: PLoS One. 2024 Mar 7;19(3):e0298601. doi: 10.1371/journal.pone.0298601 (PMC10919601; doi:10.1371/journal.pone.0298601)
Supplement: S1 Data — (ZIP) [file pone.0298601.s001.zip › Data/地级市Z_F工作报告.zip!/╜¡╦╒/╒≥╜¡╩╨/└┤╫╘╣1⁄2╓┌║┼í╛┬φ┐╦╩2╛▌═°í┐_2018.docx]

各位代表：

　　现在，我代表镇江市人民政府向大会作工作报告，请予审议，并请各位政协委员提出意见。

　　2017年工作总结回顾

　　刚刚过去的一年，是党的十九大胜利召开的喜庆之年，也是本届政府履职的第一年。面对宏观环境复杂多变、困难挑战显著增多的形势，在省委、省政府和市委的坚强领导下，我们团结和依靠全市人民，自觉践行新发展理念，扎实推进“两聚一高” (1)，统筹抓好改革发展稳定各项工作，较好完成了市八届人大一次会议确定的目标任务。预计实现地区生产总值4100亿元，增长7.2%；一般公共预算收入284.3亿元，同口径增长3.3%；固定资产投资2700亿元，增长4%；社会消费品零售总额1370亿元，增长10.8%；城乡居民人均可支配收入分别达45350元、22700元，增长8.6%、8.5%；完成省下达的节能减排约束性指标。

　　过去的一年，我们坚持“产业强市、对标苏南”，项目建设在升温。牢牢抓住实体经济这个根本，办理落实市人大1号议案(2)、市政协重点提案，全力以赴抓项目、促转型，产业发展实现量质齐升，完成工业应税销售3900亿元，同比增长15%。

　　围绕产业强市，推进了一批重大项目。健全重大项目挂钩联系、跟踪推进机制，加大对重大项目支持力度，强化土地、资金、人才、服务等保障，27个省级重大项目、180个市级重点产业项目超额完成年度投资计划，亿元以上新开工项目324个，完成投资417亿元。圣晖莱薄膜太阳能、北汽新能源汽车、尼桑动力锂电池等一批投资量大、带动力强的先进制造业项目落户。新增上市挂牌企业31家、股权融资16.3亿元，发行企业债券340.5亿元。

　　围绕产业强市，开展了一批主题招商。成功举行江苏发展大会“镇江行”合作恳谈会，举办北京央（国）企恳谈会、上海创新发展恳谈会、深圳现代服务业恳谈会等系列招商活动，先后与江苏移动、江苏电信、江苏电力、软通动力以及12家省级金融机构签订战略合作协议，全年新签约亿元以上项目149个，制造业项目利用外资占比同比提高13.7个百分点。

　　围绕产业强市，建成了一批创新载体。高新技术产业产值占规模以上工业比重49.3%，荣获首批国家知识产权强市称号。举办中国江苏•大院大所合作对接会镇江行、2017中国镇江首届创新创业人才博览会等活动，新增国家级孵化器4家、国家级众创空间2家、国家级高新技术企业191家，创成国家级公共实训基地和国家级高技能人才培训基地，大全集团获得国家科学技术进步一等奖。新增长江学者等国家级人才20人，新增本科以上人才1.69万人。

　　围绕产业强市，壮大了一批特色产业。扎实推进“3+2+X”产业链(6)，12条产业链销售占工业销售比重达51%。跻身“中国制造2025”国家级示范区，成立全省首家军民融合产业联盟，创成国家消费品工业“三品”(7)示范城市，新增4个省级高端装备制造特色示范基地、7个省级智能车间，99家企业进入省级“单打冠军”(8)储备库。创建国家全域旅游示范区，理顺三山(9)南山管理体制，推进恒大童世界、三山旅游服务基地等项目，开展“美丽镇江乡村游”系列活动；41个重点文化产业项目完成投资104亿元，成功举办2017长江国际音乐节和市第三届文化创意设计大赛。新增省级农业龙头企业10家，被授予“中国江蟹之乡”称号，“丁庄葡萄”获批国家地理标志保护产品；承办2017海峡两岸（江苏）名优农产品展销会，农业科技项目推介对接会取得圆满成功。

　　过去的一年，我们坚持聚焦重点、破解难题，工作成效在提升。坚持问题导向，统筹推进，精准发力，不断增强工作的针对性、有效性。

　　在推进城乡建设上，加大“统筹规划、规划统筹”力度，城市总体规划修改获国务院批准，出台《精美镇江建设规划》，入选全国“城市双修”(10)“城市设计”试点城市，完成历史文化名城保护、义务教育布局等规划修编。规范土地市场，合理安排土地供应数量和节奏，严格执行经营性用地招拍挂制度，营造公开公平公正的市场环境，城市价值得到应有体现，房地产市场得到有效调控。推进城建重点工程，改造危房、空斗墙房屋2.6万平方米，海绵城市建设试点任务基本完成；连淮扬镇铁路镇江段全线无障碍施工，电力路提升改造如期完成，丁卯桥路快速化改造桥梁主线贯通，镇江火车站南北一体化改造顺利实施；3家特色小镇入选省级首批创建名单，3家风情小镇入选省级培育名单。抢抓宁镇扬一体化发展机遇，加大对接争取力度，9个合作项目扎实推进，宁句轨道交通签署共建协议。调整优化高校园区规划、建设方案，镇江高专新校区交付使用。建成2个省级历史文化名村、1个市级美丽宜居镇、12个美丽宜居村，完成97条、210公里农路提档升级，改造农桥13座。

　　在深化改革开放上，扎实推进供给侧结构性改革，53个去产能项目全部完成，水泥、钢铁产能分别压减90万吨、60万吨。深入推进“放管服”改革(11)，“e办事”审批服务平台(12)正式运行，“3550”改革(13)目标全面落地，不见面审批（服务）比例达83.5%、居全省前列；新开业各类市场主体6.47万户。加快推进农村各项改革，农村土地确权登记颁证、农村集体资产清产核资全面完成，农村集体经营性资产股份制改革试点扎实推进；开展“三治理一挂牌”(14)工作，农村产权交易、农村“三资”(15)管理、涉农资金阳光监管、扶贫工作阳光监管四个平台建成使用；“农综社”(16)试点改革经验在全省推广。推进港口管理体制改革，参与省港口集团出资组建，成立镇江港口发展集团。抢抓“一带一路”(17)机遇，进出口贸易平稳增长，13家企业入选省国际知名品牌；与法国贝尔热拉克市和芬兰瓦萨市建立友城关系，成功举办印度文化周、镇江与日本仓敷市结好20周年等系列活动。

　　在保障改善民生上，十件民生实事全面完成（见附件二）。新增城镇就业8.2万人，城镇登记失业率1.9%，建成全省首家创业大学。教育现代化“双创”(18)占比达96%，建成30所智慧校园，高考成绩取得新突破。成为全国公立医院改革示范市，康复联合病房、全-专联合门诊建设全面推进，全省首家就医移动支付平台上线运行。深化文化市场综合执法改革，成为全省首批书香城市先进市。南徐片区社区商业“倍全模式”(19)深受市民欢迎。网络社会组织发展经验获中央网信办肯定。成功举办第二届江苏航空体育旅游季、市第十五届运动会，镇江籍运动员在第十三届全运会上勇创佳绩，世业洲跑步线路入选2017江苏最美跑步线路。居民消费价格涨幅2%。城乡基本社会保险覆盖率98%以上，城乡低保标准统一提高到每人每月655元，新建老年人日间照料中心7个、老年人助餐点114个。61个经济薄弱村达标、3200多户农村低收入人口脱贫。回民公墓一期建成。江苏佛学院开班办学。新建放心粮店29家。“质量强市”建设、“放心餐桌”工程(20)、食品安全示范城市创建稳步推进。

　　在解决积存问题上，认真梳理“四集团一中心”(21)闲置房产，制定化解方案，盘活率达53%。梳理规模以上工业企业“两证”(22)未办理等问题，协调解决率达43%。按照“有效解决存量、坚决杜绝增量”原则，开展违法用地专项治理，违法用地整改率达40%。加大违法建设整治力度，拆除新生违法建设891处。理顺医保管理体制，优化职工医保统账结构，整合城乡居民医保，推进医保反欺诈“亮剑”行动，一举扭转近年来医保统筹基金赤字状况。稳妥推进韦岗铁矿依法破产、联合水泥自主清算工作，完成职工分流安置。句容市生活垃圾焚烧发电厂投入使用，有效缓解“垃圾围城”矛盾。按照依法合规要求，稳妥处理西荷花塘地块、高铁南站出让地块、镇江新区汽配城、西口门砂石码头整治等问题，进一步营造亲商、爱商、安商的环境。

　　在防范化解风险上，制定地方政府性债务化解方案，实行债务总量、增幅、结构、成本“四控制”，政府性债务管理领导小组实体化运作。开展互联网金融清理整顿，严厉打击非法金融、非法传销活动，推动担保破圈解链，成功处置“8•24”涉稳事件。推进安全生产大排查大整治，持续加大监管执法力度，有效化解各类矛盾和安全隐患，全市未发生较大以上有影响的生产安全事故，事故起数、死亡人数实现“双下降”。圆满完成安保维稳重大任务，再获“全国社会治安综合治理优秀地市”殊荣。

　　过去的一年，我们坚持“生态优先、特色发展”，环境保护在加强。树立“绿水青山就是金山银山”的理念，以“263”专项行动(23)为抓手，坚决打好污染防治攻坚战，加强生态环境治理与保护。

　　坚决整改。以环保督察反馈问题为重点，建立常态化明查暗访、媒体曝光与跟踪督办工作机制，保持铁腕治污高压态势，依法关闭违法违规企业80家，关停化工企业53家，削减煤炭消费量102万吨，中央环保督察组交办事项均按序时推进。

　　综合整治。扎实开展长江“共抓大保护”行动，干流断面水质保持П类，长江非法采砂、江上过驳等专项整治行动取得阶段性成效。河长制实现全覆盖，完成金山湖、光明河、玉带河生态修复等7项重点工程；63项水污染防治项目全部完成，关闭、搬迁禁养区内养殖场795家，20个省级以上考核断面水质达标率80%，5个县级以上集中式饮用水源地水质达标率100%。330项年度大气污染防治项目全部完成，火电机组实现超低排放改造全覆盖，全市PM2.5平均浓度52微克/立方米，空气优良天数达标率71.5%。出台《镇江市土壤污染防治工作方案》，推进农业用地土壤污染详查工作。

　　有效整合。以低碳城市建设为抓手，整体推进低碳产业、低碳交通等“九大行动”(24)。成功举办2017国际低碳（镇江）大会，成为全国首家“国家低碳计量试点城市”；应邀参加第23届联合国气候变化大会，并举办“中国角”镇江主题边会，进一步扩大了低碳镇江的影响力。6家企业创成国家首批绿色工厂(25)；新建向家门等一批街头绿地；完成成片造林面积1.12万亩，林木覆盖率达25%；自然湿地保护率达52.3%，赤山湖创成国家湿地公园。

　　过去的一年，我们坚持真抓实干、务求实效，内生力量在汇聚。强化以实干创实绩、以实绩论英雄的工作导向，凝心聚力谋发展，心无旁骛抓落实。

　　竞相发展的氛围越来越浓。丹阳市以全省第一、全国第二的佳绩创成全国文明城市；成功发放全国第一份施工许可电子证书；创成全国首批“四好农村路”(26)示范县、省级农业示范园；南师大中北学院实现首批招生。句容市全力打造“江苏硅谷、生态农业、全域旅游”三张名片，一般公共预算收入增幅居全省前列，“南京大学•句容国家级创新创业示范园”开工建设。扬中市获批国家知识产权强县工程试点县、高比例可再生能源示范市和全国城乡交通运输一体化创建示范县；创成首批省级食品安全示范城市。丹徒区科技产业园正式开园，世业镇成为首批省级健康镇，永茂圩等3个村入选全省首批特色田园乡村建设试点。京口区中林（镇江）生态产业城木业先进制造区开工建设；成为中国物流金融峰会永久会址；承办2017中国学前教育年会。润州区签约韦岗旅游康养产业、启迪协信镇江低碳智创园项目；成立“镇江中德职业教育中心”，教育现代化建设水平综合得分全省第一。镇江新区成功落户江化微、奥音科技等一批重大项目，力信锂电池二期、汇鸿冷链物流基地等项目竣工投产；成为全省开发区“放管服”集成改革示范先行区；国家级开发区综合考评位列全省第六，较上年提升6位。镇江高新区实体化运作，中集车辆挂车租赁总部、量子信息产业园、创业黑马科技运营项目落户；成立全省首家科技企业融资路演服务中心。

　　服务发展的作风越来越实。扎实推进“两学一做”(27)学习教育常态化制度化，认真贯彻中央八项规定和省委、市委十项规定，加大督查问责力度，持续整治“庸懒散”问题。发扬“店小二”精神(28)，深入开展“暖企惠民”大走访，帮助企业和群众解决实际问题，市企业网络服务平台获评国家级中小企业公共服务示范平台。积极构建“亲清”政商关系，提升行政服务效能，推进行政权力网上公开透明运行。深化法治政府建设，推进学法长效机制建设，完善规范性文件和重大决策合法性审查机制，认真落实“三重一大”(29)决策制度，建立市级政府投资项目管理办法。政府系统承办人大代表建议234件、政协提案373件，满意和基本满意率100%。各部门改进作风、优化服务，政府法制、侨务、行政学院、人口计生、民族宗教、机关事务等工作取得新成绩，编制、档案、地方志、防震、气象、供销、科普、老龄、妇女儿童、关心下一代、残疾人、红十字等事业取得新进展，国家安全、国防教育、人民武装、双拥、人民防空、援藏援疆援陕等工作取得新成效。

　　共谋发展的合力越来越强。在市委的坚强领导下，市人大、市政协依法履行监督职能，有力推动政府依法行政、科学施政，让我们倍受鞭策和鼓舞；离退休老干部胸怀大局、心系发展、奉献余热，让我们倍加感动和钦佩；各民主党派、工商联、无党派人士以及各社会团体积极出谋划策、献计出力，让我们倍添信心和力量；广大基层干部群众为我们鼓劲加油，让我们倍感振奋和激励。在此，我谨代表市人民政府，向全市人民，向各位人大代表、政协委员和离退休老同志，向市各民主党派、工商联、无党派人士、人民团体和社会各界，向驻镇人民解放军、武警官兵和人民警察，向垂直部门和驻镇单位，向所有关心支持镇江发展的海内外朋友，致以崇高的敬意和衷心的感谢!

　　在肯定成绩的同时，我们也清醒地看到，全市经济社会发展中还存在一些问题和短板：一是受宏观经济下行、财税政策调整、消化过高基数、淘汰落后产能等因素影响，地区生产总值、一般公共预算收入和固定资产投资三项指标未能完成人代会确定的目标。二是产业发展不充分，形成产业链集聚的重大项目不多，新开工项目、新增长点项目的支撑作用不强。三是居民收入、企业利润、财政收入“三个口袋”有待进一步充实。四是基础设施还有短板，市域快速路网体系还未健全，农村基础设施还需加强。五是生态保护、污染防治、节能减排工作压力很大，仍需持续用力。六是防范金融风险、化解地方政府性债务的力度需要进一步加大。七是医疗、教育、养老等公共服务不平衡不充分问题较为突出。此外，政府机关少数同志不敢担当、不愿作为的状况仍然存在，政府工作人员的改革意识、服务意识、担当意识需要进一步锤炼。对这些问题，我们将高度重视，切实加以解决。

　　2018年工作总体要求

　　2018年是贯彻落实十九大精神的开局之年，是改革开放40周年，是决胜全面建成小康社会、实施“十三五”规划承上启下的关键一年。

　　按照市委七届六次全会的部署，今年政府工作的总体要求是：全面贯彻党的十九大精神，以习近平新时代中国特色社会主义思想为指导，认真落实中央经济工作会议和省委省政府以及市委的决策部署，牢牢把握稳中求进工作总基调，坚持以人民为中心的发展思想，坚定贯彻新发展理念，扎实推动高质量发展，深度对标苏南，深化改革创新，以供给侧结构性改革为主线，统筹做好稳增长、促改革、调结构、惠民生、防风险各项工作，为高水平全面建成小康社会、奋力开创新时代镇江发展新局面打下坚实基础。

　　今年经济社会发展的主要预期目标是：地区生产总值增长7%左右；一般公共预算收入增长5%左右；固定资产投资与上年持平；社会消费品零售总额增长10%；城乡居民人均可支配收入与经济增长同步；全面完成省下达的节能减排约束性指标。

　　做好今年工作，必须勇于解放思想，善于抢抓机遇，强化创新引领，注重系统提升，坚持底线思维，把发展的步子迈得更稳，把发展的质量提得更高。

　　——敏锐把握机遇。机遇是启动发展的“钥匙”。从宏观经济看，我国经济由降趋稳，稳中求进工作总基调成为经济工作的方法论和治国理政的重要原则，积极的财政政策取向不变，稳健货币政策保持中性，为我们做好各项工作提供了方向指引。新发展理念正在落地生根，经济正在向高质量发展转型，我们转得快、转得早，就有可能“变道超越”。从现实角度看，宁镇扬一体化效应已经显现并正在不断释放，我们要充分发挥镇江在成本、要素、空间等方面的优势，加大产业集聚、人口导入、人才引入的力度，争取溢出效应的最大化。

　　——坚决战胜困难。纵观国际形势，全球经济进入深度转型调整期，逆全球化和贸易保护主义抬头，不稳定不确定因素明显增多；发达国家再工业化，发展中国家加速工业化进程，我国制造业正面临“双重挤压”。从国内看，传统产能过剩、劳动力成本上升、资源环境约束趋紧等问题凸显。从我市看，一些长期积累的深层次问题，解决起来还需要一个过程。我们一方面要把困难估计得充分一点，另一方面要拿出行动的勇气，有的放矢，赢得主动。

　　——保持战略定力。“产业强市、对标苏南”正在成为干部群众的行动自觉。深度对标苏南，不是“数字对标”“口号对标”，而是理念、眼界、思路、行动上深度对标，既不为GDP增长一时的快慢而纠结，也不为排名一时的先后而焦虑。宁可发展速度慢一点，也要发展质量高一点；宁可增长速度低一点，也要转型步伐快一点；宁可政府开支紧一点，也要群众实惠多一点。脚踏实地、久久为功，一定能积小胜为大胜。

　　——坚定发展信心。镇江有着悠久的工商文化传统，涌现出陈光甫、陆小波、柳传志等一批工商巨子，这种传统基因完全能够在传承中发扬光大。镇江有着得天独厚的自然禀赋和人文底蕴，“有山有水有故事”(30)，完全能够转化为区域竞争的比较优势。镇江有着一支在市场经济大潮中成长起来的企业家队伍，完全能够勇立潮头、奋勇争先。镇江有着不甘落后、务实进取的干部队伍，有着勤劳质朴、聪明智慧的人民群众，完全能够谱写无愧于时代的新篇章。

　　突出重点 狠抓关键

　　做好今年工作，围绕高质量发展，着力在六个方面狠下功夫，努力在新时代、新征程中展示新形象、实现新作为。

　　一、构筑产业发展新高地

　　改革开放以来，镇江曾经创造了乡镇企业、港口型经济的辉煌；今天，必须更加重视实体经济，坚定不移走产业强市之路。

　　壮大优势产业。实施高端装备制造、新材料两大主导产业三年提升计划，加快工业机器人、通用飞机、新能源汽车、高技术船舶与海工装备等领域重大装备研制赶超，力争两大主导产业销售占全市工业销售比重提升2个百分点。深度推进军民融合发展，提高先进材料供给质量，扩大新材料示范应用，建立重大技术装备和新材料协同发展机制。推动产业集聚发展，重点打造航空航天、智能电气、船舶海工等具有地方特色的地标产业。加快布局前沿产业，积极寻求与大院大所合作，力争在人工智能、增材制造、储能技术、新一代5G移动通信技术等领域取得突破。

　　提升传统产业。按照“智能化、绿色化、品牌化、服务化”要求，推动传统产业转型升级。深化制造业与互联网融合发展，实施智能制造“三百工程”(31)，新增省级以上智能工厂（车间）10家。开展工业绿色提升行动，建成1家国内领先的绿色创新中心，新增市级以上绿色工厂20家。开展企业“三同”(32)试点示范，促进“三品”战略向其他领域延伸，新增市级“专精特新”产品10个。实施工业设计提升计划，组织企业向研发设计、售后服务两端发力，延伸产品价值链；积极培育网络化协同、个性化定制、在线化增值服务、分享制造等服务型制造模式，新增省级工业设计中心1家、服务型制造示范企业2家。

　　培育领军企业。围绕龙头企业发力，促进中小企业配套，有效延伸产业链，打造更多百亿级龙头企业；培育细分领域“单打冠军”企业，新增市级“单打冠军”15家；支持企业加快建设高水平研发机构，新增授权发明专利2500件以上、省级科技小巨人企业5家。促进金融回归本源、服务实体，持续降低企业融资成本；新增上市挂牌企业26家，加强上市公司市值管理，增强价值创造、经营能力。大力弘扬企业家精神，尊重、善待、支持、服务好企业家，深入开展“暖企”大走访，既服务好外来投资的企业家，又团结好本土企业家；既充分发挥老企业家作用，又大力培养新生代企业家，引导企业家坚守实体、深耕主业、专注创新。

　　加大有效投入。以高质量的项目、高质量的投入、高质量的增量推动高质量发展，加快推进北汽新能源汽车、尼桑动力锂电池等175个市级重点产业项目，当年完成投资410亿元，坚定不移做强制造业特别是先进制造业。切实提高招商水平，开展产业链招商、规划招商和园区招商，探索采取“基金公司+咨询公司”“投行+基金”等模式，以市场方式撬动重大项目落地和产业集聚，新签约亿元以上产业项目150个。

　　二、培育驱动发展新动能

　　以历史眼光、全球视野深刻把握发展的趋势和规律，大力培育发展新产业新动能，让发展质量更高、竞争力更强。

　　强化创新驱动。抢抓宁镇扬一体化发展机遇，积极推进“一带三谷”建设，以江苏硅谷、团山睿谷、中业慧谷为重要节点，加快建设G312创新发展带，吸引更多创业企业和人才入驻，形成集聚效应。充分运用技术平台的核心作用，促进互联网、大数据、云计算、人工智能与各行业领域的深度融合，积极发展共享经济、数字经济、生物经济、现代供应链等新业态新模式。建立高新技术企业培育库，新认定国家级高新技术企业100家，新增国家级企业技术中心1家。深入实施“金山英才”计划，启动“百万校友资智回镇工程”，新增“千人计划”、长江学者等国家级人才15人，“双创计划”、科技企业家等省级人才50人。加强与驻镇高校深度合作，促进科技成果在本地转化。弘扬“工匠精神”，开展各类职业技能培训，实施乡土人才“三带”行动计划(33)，新增高技能人才1万人。

　　强化改革推动。深化供给侧结构性改革。重点在“破”“立”“降”上下功夫。坚持用市场化、法治化方式淘汰落后产能、化解过剩产能，实施40个去产能项目，“僵尸企业”(34)基本出清；持续降低企业生产经营性成本、制度性交易成本。扎实开展第四次全国经济普查工作。深入推进行政审批制度改革。推动“e办事”审批服务平台终端镇（街道）全覆盖。深化“3550”改革，落实登记便利化措施，推进证照分离改革试点工作；全面推进房产交易与不动产登记两中心整合，简化操作流程，提高办事效率；推进施工许可证发放环节流程再造，拓展区域评估范围，优化“互联网+多联合一” (35)集成应用。积极推进国资国企改革。整合国有金融、类金融机构(36)，做大做强金融产业；加快国有平台公司市场化转型，完善市属产业集团运行机制。深化农村综合改革。全面推进农村集体产权制度改革，支持丹阳全域开展农村集体资产股份制改革，实现农村集体资产保值增值；推进农村土地“三权分置”(37)改革；加强涉农资金整合与监管，切实保障农民财产权益。

　　强化开放带动。加大制造业利用外资力度，力争实到外资6.5亿美元。鼓励重点企业实施跨区域、跨行业并购重组，实现全球配置资源，形成规模优势，增强辐射能力。加快货物贸易优化升级，稳住欧美日等重点出口市场，积极开拓“一带一路”和自贸协定签约国市场，争创省级内外贸结合市场试点；建设线上线下外贸综合服务平台，为中小外贸企业提供全流程服务；加快建设中林木业等进口集散中心。举办2018年国际友城大会、与韩国益山市结好20周年纪念活动，开展“第四届东盟文化之旅”，设立“国际友城之窗”，拓展友好城市交流空间。

　　强化园区提升。开发园区是经济发展先行区，是改革创新主阵地。以“一特三提升”(38)为抓手，编制完成开发区特色主导产业专项规划，加快产业集聚，厚植特色优势。国家级经济开发区、国家级高新区加快转型升级，实现争先进位；20个产业“三集”(39)示范园区、8家市级特色小镇完善功能，提升产业承载力；支持丹阳创建国家级经济开发区、句容创建国家农业高新区和省级高新区、扬中创建国家级高新区、丹徒创建省级高新区，推动镇江高新区中挪海工产业园上升为国家间合作园区。加快产业园区向现代园区转型，合理布局公共服务配套设施，建设金融、物流、信息、科技等生产性服务设施，将开发区打造成投资创业园、创新集聚区、生活宜居城。

　　强化资源整合。挖掘存量资源，加强精细管理，确保各类资源发挥最大效益。整合港口岸线资源。发挥“江海河联动、港产城融合、集疏运一体”的优势，支持港口发展集团整合资源、优化布局，做大物流产业。整合风景名胜资源。整合三山风景区资源，建成三山旅游服务基地，打造长江沿线最美旅游路线。推进南山鹤林景区建设，推动南山西入口景观改造提升，打造莲花洞景区杜鹃花谷；推进南山东入口整治，打造八公洞景区。推进恒大童世界等旅游项目，做好旅游风情小镇创建工作，赤山湖、江心洲橘江里等创建4A级景区，茅山湖创建国家级旅游度假区。盘活闲置房产资源。全面完成“四集团一中心”闲置房产盘活任务，完善政企联动招商机制，确保招商有质量、盘活有效率；加强统筹规划，合理布局商业设施，壮大城市楼宇经济。盘活存量土地资源。推进工矿废弃地复垦开发；落实闲置土地联动处置机制，盘活低效土地；完成违法用地整改任务，建立协调联动机制，坚决遏制增量发生。

　　三、构建联动发展新格局

　　加强统筹协调，推进区域协同、智慧互联、城乡统筹发展，拓展城市发展的空间。

　　外联一体。抢抓宁镇扬一体化发展机遇，推进连淮扬镇铁路镇江东站、镇江新区站建设；开工建设宁句轨道交通、南沿江城际铁路以及镇江长江大桥南、北接线高速公路；启动312国道宁镇段快速通道建设，实现镇江到南京快速通达；开展镇江扬州过江第二通道前期研究；推进京沪高铁镇江南站综合客运枢纽交通功能优化改造，加密宁镇城际铁路点对点发车班次，探索实现公交化运营。系统深入研究镇江综合交通枢纽的规划、布局和功能，放大其带动效应。

　　内聚成核。研究240省道南延至122省道、122省道快速化改造方案，构建“一横一纵”骨干快速路网体系，促进主城区与丹阳、句容、丹徒紧密联结，带动镇江南部腹地的发展；建成镇江新区至丹阳高速公路；推进新312国道东延至规划233国道段，西延至高资通江路，完成中心城区外环闭合。研究谷阳路铁路桥重建方案，力争在连淮扬镇铁路通车调试前建成。

　　智慧互联。紧紧抓住“互联网+”、大数据、智慧城市建设等国家战略，以“善政、兴业、惠民”为目标，构建“一中心、一平台、多应用”的智慧城市体系，着力打造智慧化“城市大脑”。完成大数据中心建设，实现智慧应用系统互联互通。建设城市综合运行监管平台，提供风险监测、预警分析、综合调度、应急处置以及成果展示、技术体验等功能。推进智慧信用、智慧旅游、市民卡工程、工业云平台等应用，逐步实现政府决策科学化、社会治理精准化、公共服务高效化。

　　品质提升。树立“精明增长”“紧凑城市”理念，高质量编制各类规划，推进“精美镇江”建设。优化主城区“一核四区”(40)、中心城区“一体两翼”(41)格局，南徐片区、官塘片区、高校园区添内容、添功能、添人气，推动形象出新、功能变优。实施谷阳路改造、长香路拓宽改造等项目，打通汝山路南段、宗泽路中段等“断头路”，畅通城市“内循环”。推进城市治理与服务十项行动(42)，结合“城市双修”要求，统筹推进棚户区改造和老旧小区有机更新。启动棚户区改造三年行动计划，实施10个片区改造项目，完成棚改任务6000套；实施老旧小区改造三年行动计划，采取“集中成片、点面结合”方式，运用“共同缔造”理念(43)，确保三年全面完成主城区145万平方米改造任务。

　　乡村振兴。按照“产业兴旺、生态宜居、乡风文明、治理有效、生活富裕”的要求，建立健全城乡融合发展体制机制和政策体系。深化农业供给侧结构性改革，实施质量兴农战略，壮大优质粮油、高效园艺等五大特色产业，发展休闲农业、乡村旅游、农村电商等新业态，新增4个省级以上休闲农业示范点；培育新型职业农民7000人、农业科技示范户3000户以上。推动农村基础设施提档升级，完善水利、电力、通信等设施，完成230个规划发展村庄生活污水治理任务，推进扬中三茅主江堤复建、丹阳新孟河延伸拓浚和九曲河整治，新建高标准农田12万亩。推进乡村绿色发展，实施“生态河湖行动计划”(44)，有序按流域整治乡村水环境；加强农村突出环境问题综合治理，严格控制和治理农业面源污染，秸秆综合利用率保持95%以上。创新乡村治理体系，加强农村基层基础工作，完善村级治理架构，支持各类社会组织参与农村社区治理与服务。传承发展提升农村优秀传统文化，加强对古村落、民间技艺、农耕文明的保护利用，培育文明乡风、良好家风、淳朴民风，不断提高乡村社会文明程度。

　　四、打造特色发展新亮点

　　以系统化思维推进生态文明建设，针对短板、弱项，下决心，出重拳，加大力度，加强监管，擦亮生态名片，形成鲜明特色。

　　深化一个专项行动。继续开展“263”专项行动，坚持源头管控、精准治理，坚决打赢污染防治攻坚战。深化减煤减化工作，削减煤炭消费量87万吨，关停转移化工企业42家，化工企业入园率达到50%。深化“河长制”(45)“断面长制”(46)监管模式，完成御桥港等5个黑臭水体整治任务，推进控源截污、雨污分流、管网改造、河道清淤，加快构建水循环体系，太湖流域重点断面水质达标率90%以上。建立土壤环境质量数据库，有序推进土壤污染治理与修复。开展第二次全国污染源普查工作，建成焚烧灰渣处置场二期工程。加强长江镇江段水质保护、水文监测，提高江堤防护标准；全力抓好非法码头和排污口整治、沿江化工园区和危化品专项治理等工作，加强突发环境事件风险防控。

　　开展两项集中打击。集中打击长江非法采砂，坚持“水打”与“陆治”相结合、日常监管与专项打击相结合，加强明查暗访，强化部门协调，对重点水域开展全天候巡查，规范水上过驳市场经营行为，建立完善“三无”船只(47)、浮吊、码头监管的长效机制，确保监管有力、打击有效。集中打击非法开山采石，全面关停非法轧石场、洗石场、石灰窑等企业，加快拆除关停企业的厂房设备，开展不间断、全天候、常态化监控，坚决防止死灰复燃。进一步压实责任，加强整顿，切断利益链条。

　　突出三个片区整治。制定整治计划，形成项目清单，分步推进，久久为功。西南片区突出粉尘治理。抓住韦岗铁矿破产处置、联合水泥自主清算的有利契机，对废弃采石宕口、关闭取缔的轧石厂、石灰窑等地块，宜垦则垦，宜林则林，完成复垦复绿，启动韦岗温泉康养小镇规划建设。东部片区突出异味整治。所有化工企业完成挥发性有机物污染防治系统建设，从源头上减少异味废气排放；建立空气质量网格化监测系统，加强动态监测预警。研究推进城东垃圾填埋场生态修复，完成一夜河控源截污、河道拓宽、边坡治理及景观建设，同步实施支流河道清淤、沿线排口整治。高资片区突出循环改造。实施产业绿色转型，推动园区循环化改造，建立循环经济产业链，支持丹徒经济开发区争创国家级循环化改造园区。

　　推进四项重点任务。推动低碳城市建设。在全市上下开展低碳城市建设工作，树立“低碳发展，我有责任”的理念，推行低碳发展和低碳生活；继续实施低碳“九大行动”，推进扬中“近零碳岛”、低碳产业技术研究院、低碳小镇建设，精心举办2018国际低碳（镇江）大会。完善生态补偿机制。探索建立多元化生态保护补偿机制，逐步扩大补偿范围，合理提高补偿标准，推进环境污染第三方治理，有效调动全社会参与生态环境保护的积极性。构建能源供应体系。研究全市能源结构调整，研究东部地区热源点建设，逐步推动燃煤大机组供热和清洁能源的互联互通，支持开发区加快建立循环型、梯级型、智慧型能源供应体系。建立环境监管体制。完成环境监管体制调整，实行网上受理与实地查证联动、环境监察与环境执法联动，构建信息化的环境监察流程。加强环保信用监管，推动企业环保信用评价结果在行政许可、采购招标、银行贷款等工作中广泛应用，构建“一处失信、处处受限”的失信惩戒格局。

　　五、提升惠民发展新成效

　　坚持把人民对美好生活的向往作为奋斗目标，更好地满足人民日益增长的美好生活需要。

　　推动就业创业。完善创业扶持政策，加大对大学毕业生、企业骨干、返乡创业者等重点创业人群的跟踪服务，扶持创业7000人以上，创业带动就业3.5万人。构建“创业大学+驻镇高校+辖市区分院+企业基地”四维创业载体，打造培训教育、创业孵化、融资服务等各类平台，力争创成省级创业示范基地2家、市级创业示范载体5家。弘扬创业文化，开展“企业家论坛”“百名专家进百企”等活动，鼓励更多专业技术人员投身创业，强化尊重劳动、尊重知识、尊重创造的价值取向，让一切诚实劳动者得到激励，让所有依法纳税人受到尊重。

　　深化精准脱贫。深入实施精准帮扶工作，强化“阳光扶贫”监管平台的应用和管护，瞄准贫困人口和弱势群体，壮大村级经济实力，促进农民增收致富；全面建立社会救助“一门受理、协同办理”机制，加强动态监测，实现精准施保、分类保障、应保尽保、应退尽退；搭建困难群众“救急难”绿色通道，构建政府救助、慈善补充、群众互助的多元化救助体系。

　　优先发展教育。立足办好人民满意的教育，调整优化义务教育学校布局，完善“规范+自主、全面+特长”育人模式。加强幼儿园建设与城镇化、二孩政策的衔接，优化早教托育社会化服务体系。深化校长职级制、教师“县管校聘”(48)等综合改革，加强师德师风建设、业务能力培训，提升师资队伍水平。

　　建设健康镇江。实施“三医”联动(49)综合改革，合理配置医疗资源，推进市医疗集团实体化运行，实施基层医疗机构示范创建、特色专科培育、中医药能力提升“三大工程”，推进医保支付方式、职工基本医疗保险统账运行方式改革，升级医保智能监控系统。实施健康管理示范乡镇（街道）三年行动计划，深入推进世业镇健康小镇建设，培育健康养老产业，打造“居者乐业、游者向往”的长江健康第一岛。

　　繁荣文化事业。创建“国家级公共文化服务体系示范区”，推进文化设施建设；开展“文心”系列文化惠民活动，实施“书香镇江”数字化工程，启动国家级代表性传承人抢救工程。推进古运河文化带建设，建设大龙王巷、伯先路历史文化街区，完成江苏电力历史博物馆一期工程，保护利用好沈括、赛珍珠、郭礼征、张鹏、陆小波等名人故居，挖掘特色街巷文化，保护好传统风貌建筑。修复天下第一泉。

　　加强体育事业。启动城乡公共体育设施三年提升计划，建设健康路市民体育公园，改造蛋山体育公园，加强文体活动广场建设；推动学校体育场馆向社会有序开放；引导足球进学校、进课堂，新增44所全国特色足球学校；开展“全民健身日”、乐在“棋”中等全民健身品牌赛事活动，承办好2018年全国青少年“未来之星”阳光体育大会等重大赛事。

　　完善保障体系。实施全民参保计划，重点抓好灵活就业、新业态就业、中小企业人员等重点群体的参保工作，主要险种参保率98%以上；加大社保基金管理，建立监测预警机制，常态化开展医保反欺诈“亮剑”行动；加强社会保障制度衔接，构建全覆盖、多层次、可持续的社保体系。推动养老服务设施优化布局，鼓励和引导社会力量兴办或运营养老服务产业，推动医养融合，加强老年人精神关怀。

　　六、强化保障发展新机制

　　牢固树立“守土有责、保一方平安”的责任意识，营造安全、和谐、稳定的发展环境。

　　创新社会治理机制。扎实开展社会治安专项行动，依法打击各类违法犯罪，构建立体化、信息化社会治安防控体系，提升群众安全感和法治满意度。深化“网格+网络”(50)治理体系，推进“政社互动”(51)“三社联动”(52)，探索社委会、业委会、物委会联合开展工作机制，促进社区、社会组织、社会工作服务融合提升。推动执法重心下移，稳妥实施三山南山风景区和镇江高新区城市管理执法体制调整。开展马路市场、占道经营、违章搭建等“八项整治”(53)，提升精细化管理水平。

　　完善公共安全保障机制。推动安全生产领域改革，建立责任全覆盖、管理全方位、监管全过程的安全生产保障体系，生产安全事故起数、死亡人数保持“双下降”。常态化整治交通违法行为，涉酒交通事故、工程运输车交通事故起数和死亡人数实现“双下降”。持续开展危化品、建筑施工、消防安全、校园安全等重点领域的专项整治行动，有效遏制重特大事故发生。

　　建立重大风险防控机制。按照“管总量、调结构、降成本、控增速、不出险”目标，规范市属产业集团融资行为，逐步将政府债务风险指标控制在合理区间。建立金融风险监测预警机制，加强对互联网金融、互联互保、社会集资的监管与稳控，坚决打击恶意逃废债；开展授信总额联合管理，支持企业续贷转贷，加快完善担保体系，审慎处置不良资产，营造良好的金融生态环境。深入推进社会矛盾和风险隐患大排查大整治，加强律师等第三方参与信访接待和化解工作，有效预防、全力化解矛盾纠纷、信访积案，建设更高水平法治镇江、平安镇江。

　　今年，将努力办好涉及公共利益、事关民生福祉的10件实事。

　　1. 公共交通。城乡公交线路新增10条、优化40条，新增150辆新能源公交车；建设环金山湖停车场、三中西侧停车场，对老小区、社会公用停车场进行升级改造，新增停车泊位1万个以上；完成98条、200公里农路提档升级，改造农桥12座。

　　2. 公共设施。新建市图书馆，改造民艺馆、博物馆并对外开放，基本完成科技馆、妇女儿童活动中心、青少年活动中心改造工程；完成中山路小学、八叉巷小学、三中、四中等学校布局调整和江南学校扩建；易地新建实验初中，原实验初中改扩建为李家大山小学。

　　3. 公共空间。全市新建街头绿地5个，市区新建体育公园5个、健身路径70条；新建、改造应急避难场所5个。

　　4. 公共环境。空气优良天数达到72%，PM2.5平均浓度同比下降5%，20个省级以上考核断面水质优Ⅲ类比例达到65%以上；实施危房改造和直管公房修缮1.8万平方米，完成10个片区、50万平方米的老旧小区改造任务；在全市354个党政机关、企事业单位和252个基层社区开展生活垃圾分类试点工作。

　　5. 公共安全。用科技手段提升城乡社会治安智能化防控水平，涉及民生类的公共安全视频监控联网共享率达到100%；全市学校、养老机构“明厨亮灶”(54)覆盖率达到85%以上，创成8家市级“放心肉菜示范超市”；危险化学品安全监管覆盖率达到100%；市突发事件预警信息发布中心正式运行。

　　6. 公共卫生。完善家庭医生签约服务，提高慢病管理水平，重点人群签约服务率达到60%；建成4个省级健康镇，新建献血屋；全市建档立卡低收入家庭中的非社会医疗救助对象，在扶贫帮困期间，参照享受社会医疗救助待遇。

　　7. 公共文化。举办全民艺术知识普及活动500场，送戏下乡500场，送电影下乡6000场，万人拥有公共文化服务设施面积新增70平方米。

　　8. 公共厕所。推进“厕所革命”(55)，全市新建、升级改造公厕50座，新建、改扩建旅游厕所60座；主城区全面消除旱厕。

　　9. 公共养老。新建5个老年人日间照料中心、100个助餐点，全市护理型养老床位占养老机构床位总数的55%以上。

　　10. 公共事务。市“12345”管理机构实体化运行，提升为民办事效能；设立市不动产登记中心京口分中心；推进网格化管理，每个网格配备1名以上专兼职网格员。

　　砥砺奋进 扎实奋斗

　　新时代开启新征程，新征程呼唤新作为。新的一年，我们将深入学习贯彻党的十九大精神，以习近平新时代中国特色社会主义思想为指导，增强“四个意识”(56)、坚定“四个自信”(57)，加快推进政府治理体系和治理能力现代化，以砥砺奋进、扎实奋斗的实绩，不辜负全市人民的期待和重托。

　　以实事求是的作风扎实奋斗。坚持一切从实际出发，按客观规律办事，把情况弄清、把工作做细、把数字搞实，坚决杜绝数字造假、统计掺水；充分考虑需要与可能，既尽力而为、又量力而行，不提好高骛远的目标，不提脱离实际的口号，说了算、定了干，干则必成，干则必精。淡化一些总量指标，注重劳动效率、资源效率、环境效率和科技贡献率，更强调质量结构效益和分配，既积极有为，又留有余地。

　　以造福人民的情怀扎实奋斗。发展的最终目的，是造福人民；造福人民是最大的政绩。进一步增强责任感和使命感，努力践行以人民为中心的发展思想，把百姓安危冷暖时刻放在心上，想群众之所想，急群众之所急，认真解决群众关心的热点难点问题，让发展更有温度，让人民有更多的获得感、满足感、幸福感。

　　以敢于担当的作为扎实奋斗。担当是全体公务人员的从政准则、成事之要，对中央和省、市委的决策部署，坚决贯彻落实；对确定的目标任务，坚决推进到位；对向人民作出承诺的民生实事，坚决如期兑现。问题在谁那里，第一时间扛起来；责任交给谁，必须不折不扣完成，不搞遥控指挥、不做甩手掌柜、不搞“击鼓传花”，以创新的办法破解矛盾困难，以行动的勇气化解各类风险。

　　以依法行政的思维扎实奋斗。坚持学法、尊法、守法、用法。政府一切工作都必须限制在法治的框架内，一切权力都必须约束在制度的笼子里，依法接受人大及其常委会的法律监督和工作监督，自觉接受人民政协的民主监督，认真听取各民主党派、工商联、无党派人士和人民团体的意见，主动接受社会和舆论监督，不断提升决策科学化、民主化、法治化水平。

　　以清正廉洁的操守扎实奋斗。纠正“四风”(58)不能止步，作风建设永远在路上。始终保持“如履薄冰、如临深渊”的自觉和警觉，扎实推进“两学一做”常态化制度化，认真开展“不忘初心、牢记使命”主题教育，严格遵守政治纪律和政治规矩，严格执行中央八项规定实施细则，坚决防止“四风”问题反弹回潮；严格落实“一岗双责”，深入推进党风廉政建设，牢固树立过紧日子的思想，加强审计监督，强化财政监管，筑牢制度“篱笆”，防范权力“越线”，树立为民务实清廉形象。

　　各位代表，一千多年前，唐代诗人王湾在北固山，写下了传颂千古的诗句：“潮平两岸阔，风正一帆悬”。站在新的历史起点上，置身于318万镇江人民之中，我们坚信，在以习近平同志为核心的党中央坚强领导下，在市委的正确领导下，全市上下砥砺奋进，扎实奋斗，一定能够创造美好的生活，实现新时代镇江高质量发展的良好开局！
